# Supplementary material for: Transcription shapes DNA replication initiation to preserve genome integrity
Source: Genome Biol. 2021 Jun 9;22:176. doi: 10.1186/s13059-021-02390-3 (PMC8188667; doi:10.1186/s13059-021-02390-3)
Supplement: Supplementary file 1 — Additional file 1: Figure S1. Experimental procedure for NAIL-seq. Figure S2. Comparison among NAIL-seq, Repli-seq, OK-seq, SNS-seq, and EdU/HU-seq in K562 or mouse primary splenic B cells. Figure S3. The landscape of ERIZs. Figure S4. Transcription disturbance relocates ERIZs in K562 cells, mSBCs, and mESCs. Figure S5. Transcription inhibition does not lead to ORC2 relocation. Figure S6. dCas9-mediated transcription blockade induces MCM5 accumulation and replication initiation. Figure S7. Collision of transcription and DNA replication initiation induces genome instability. Figure S8. The full uncropped pictures for Western blots. [file 13059_2021_2390_MOESM1_ESM.pdf]

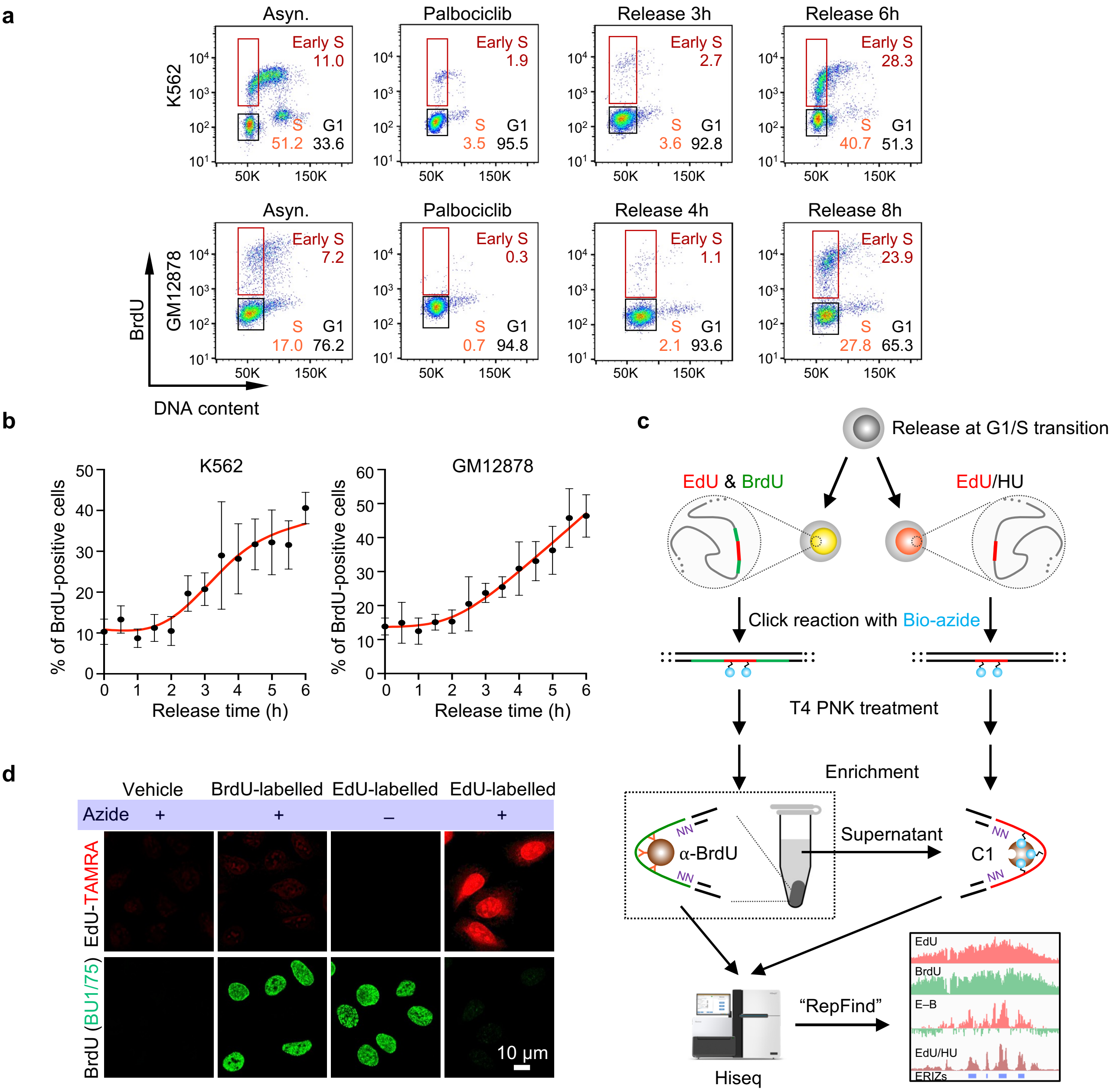

Fig. S1 Experimental procedure for NAIL-seq.

a Cell cycle analysis following BrdU and 7-AAD double staining via FACS, with or without synchronization and the following release. K562 and GM12878 cells were treated with palbociclib or vehicle for 36 hours and released into fresh medium for the indicated time. Before harvest, cells were labeled with BrdU for 30 minutes. The black gates indicate cells in the G1 phase and the red gates indicate cells in the early S phase. Asyn., asynchronized. The orange number is the percentage of S-phase cells.

b Percentage of S-phase cells following release from G1 arrest. K562 or GM12878 cells were arrested by the addition of 5 or 1 μM palbociclib, respectively, for 36 hours, and subsequently placed in fresh medium for the indicated time. Cells were incubated with BrdU for 30 minutes prior to harvest. The fluorescence microscopy results are represented as the mean ± SD of three biological replicates.

Fig. S1 Experimental procedure for NAIL-seq.

c The library preparation process for NAIL-seq. G1-arrested cells were released into the early S phase in the presence of one or more thymidine analogs. Biotinylated EdU-labeled nascent DNA was isolated by streptavidin, and BrdU-labeled DNA was purified using an anti-BrdU antibody. Enriched DNA fragments were ligated to barcoded bridge adapters, tagged by Illumina sequencing primers, subjected to Hiseq sequencing, and analyzed using the RepFind pipeline. Left panel: cells were labeled sequentially with EdU and BrdU. Right panel: cells were incubated with EdU in the presence of HU. S/N indicates the supernatant and C1 indicates the streptavidin-coupled magnetic beads.

d Detection of BrdU, EdU, and biotinylated EdU by the BrdU antibody, BU1/75. Cells were pulse-labeled with BrdU or EdU for 30 minutes and subsequently subjected to the Click reaction with TAMRA-biotin-azide or DMSO. BU1/75 was detected by fluorescence microscopy. “+” or “–” represents the presence or absence of TAMRA-biotin-azide, respectively.

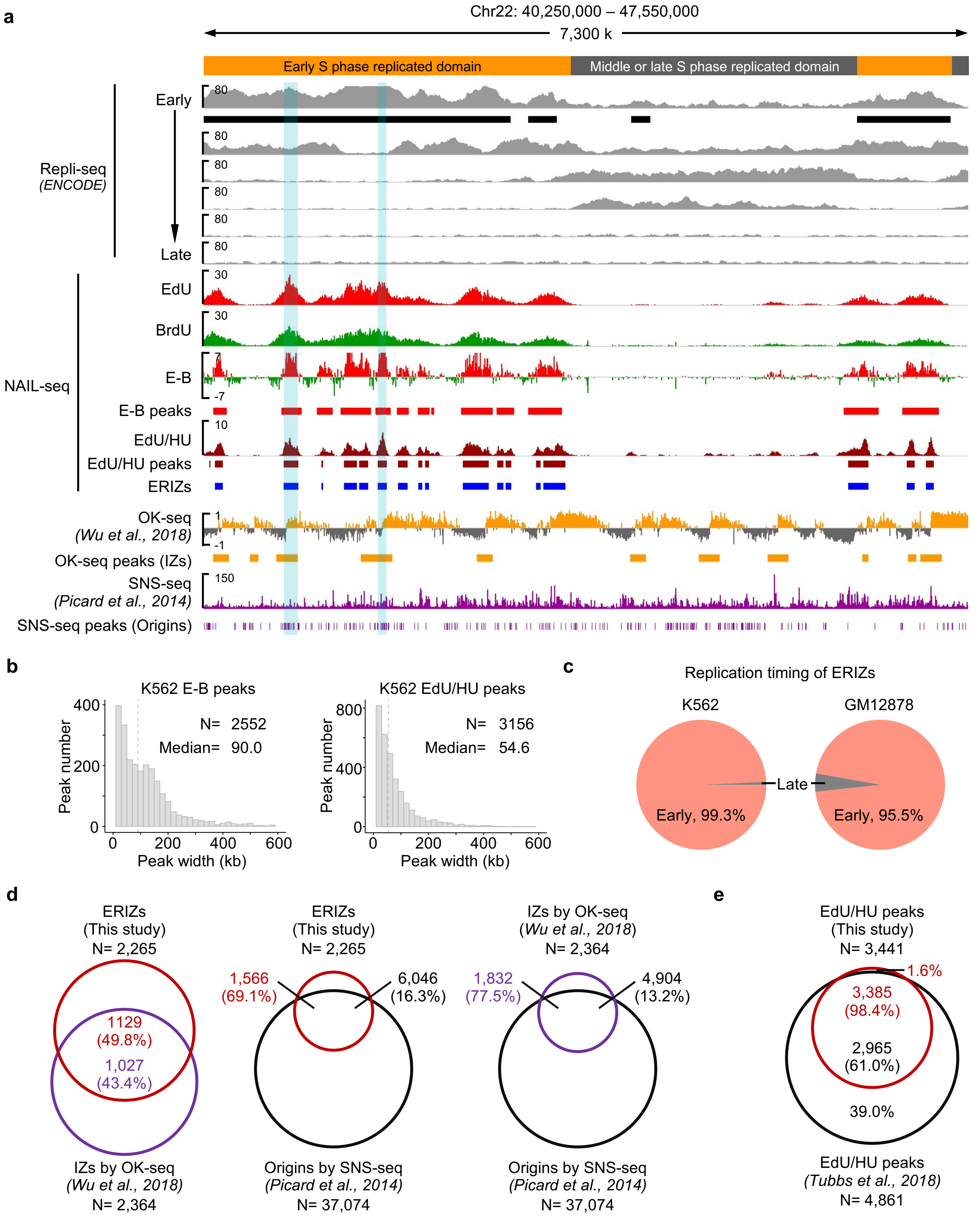

Fig. S2 Comparison among NAIL-seq, Repli-seq, OK-seq, SNS-seq, and EdU/HU-seq in K562 or mouse primary splenic B cells.

Fig. S2 Comparison among NAIL-seq, Repli-seq, OK-seq, SNS-seq, and EdU/HU-seq in K562 or mouse primary splenic B cells.

a A representative region showing the replication domains (from Repli-seq), ERIZs (from NAIL-seq), IZs (from OK-seq), and replication origins (from SNS-seq). The tracks from Repli-seq are shown in grey, and the G1 peaks (black) show the earliest replication domains. EdU-labeled regions (red) and BrdU-labeled regions (green) from the "EdU-then-BrdU" assay and the EdU/HU (dark red) signals from NAIL-seq are shown in the indicated regions. Peaks from NAIL-seq called by the "RepFind" pipeline are shown underneath. Early replication initiation zones (ERIZs) are marked in blue. The RFD signals from OK-seq are shown in orange (positive) and grey (negative). The replication initiation zones (IZs) are shown in orange. Signals from SNS-seq are marked in purple. The cyan shadow boxes highlight ERIZs containing multiple replication origins identified by SNS-seq.

b The width distributions of E-B peaks and EdU/HU peaks in K562 cells. The total number (N) and the median width of the peaks are shown in the legends.

c Pie diagram showing the replication timing of ERIZs from K562 and GM12878 cells.

d Venn diagram showing the overlapping peaks among ERIZs (red), IZs (purple), and replication origins (black) in K562 cells.

e Comparison of the EdU/HU peaks from mouse splenic B cells between the present study (red) and a previous report [15] (black).

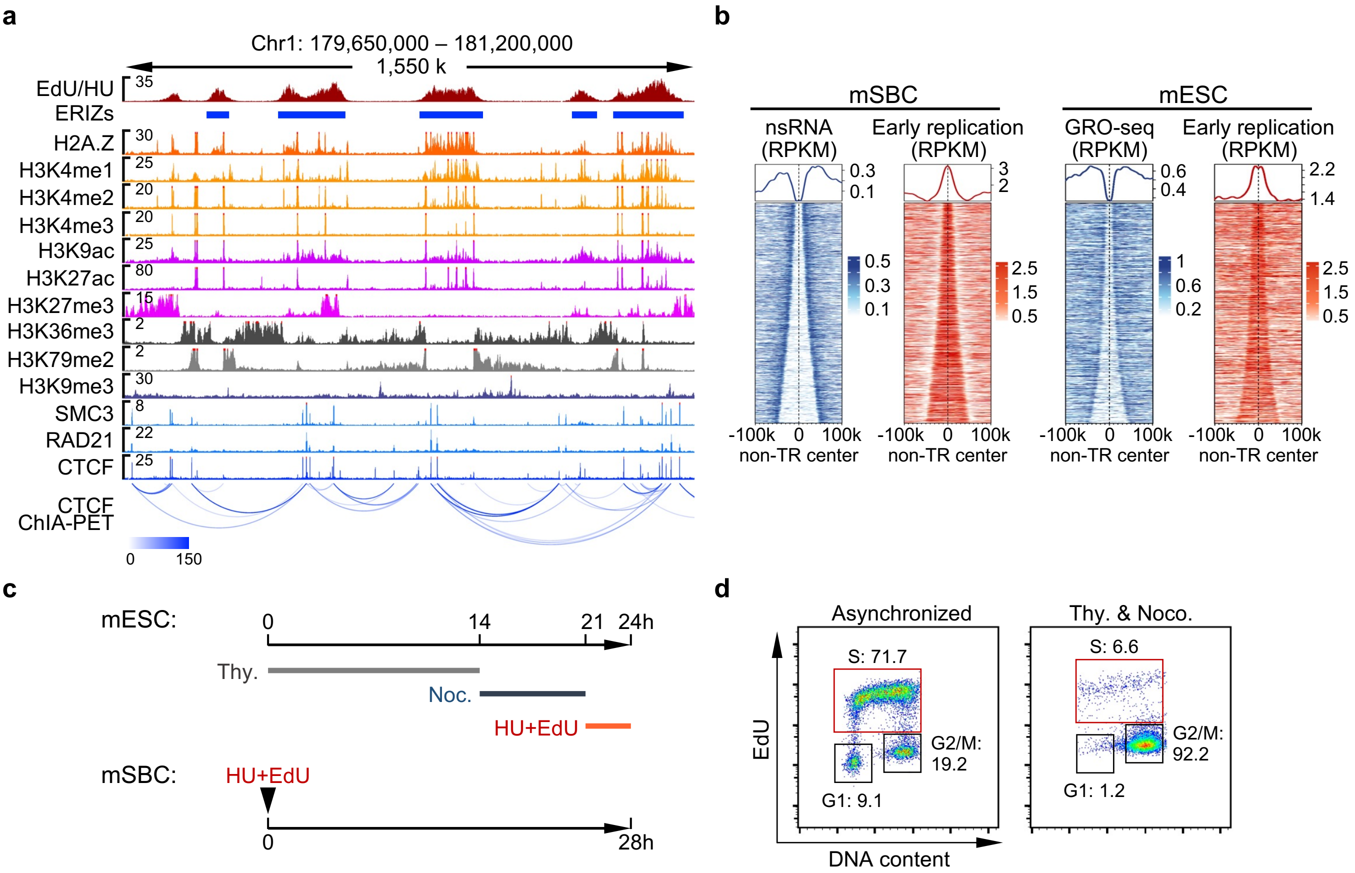

Fig. S3 The landscape of ERIZs.

a Distribution of ERIZs, histone modifications, and chromatin structural proteins within A compartments in K562 cells.

b Heatmaps of early replication initiation (from EdU/HU, red) and active transcription (blue) in mouse splenic B cells (mSBC) and mouse embryonic stem cells (mESC). The non-transcribed regions (non-TRs, see methods section for detail) are ranked by width and centered on the midpoint flanked by two transcribed genes in each cell line. The non-TRs in A compartments, 20−100 kb in width, are displayed. Each line represents a non-TR.

c Schematic showing the labeling strategy of EdU/HU for mESCs and mSBCs.

d Cell cycle analysis of asynchronized and synchronized mESCs by EdU and 7-AAD.

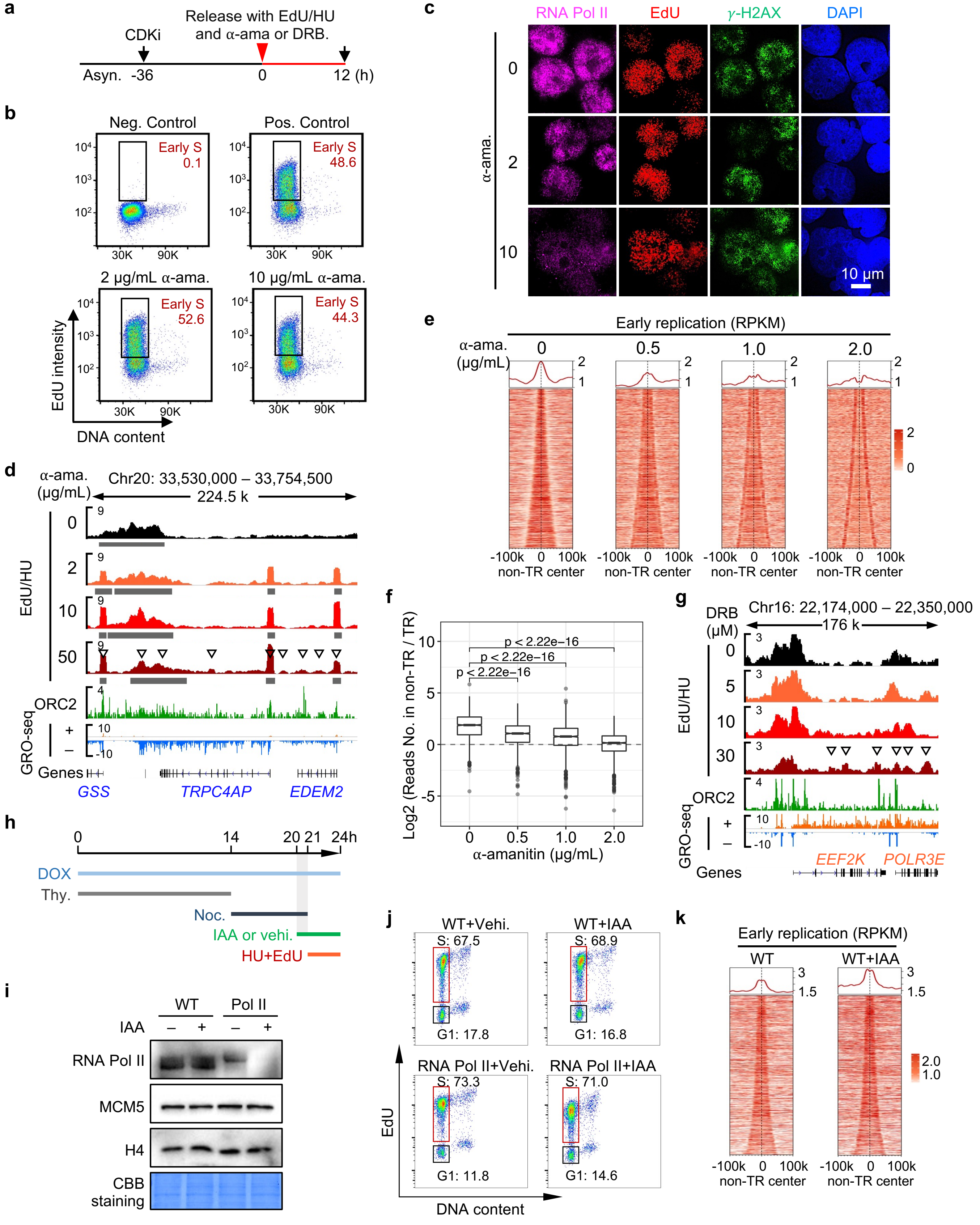

Fig. S4 Transcription disturbance relocates ERIZs in K562 cells, mSBCs, and mESCs.

Fig. S4 Transcription disturbance relocates ERIZs in K562 cells, mSBCs, and mESCs.

a Schematic showing treatment of K562 cells in the presence or absence of α-amanitin or DRB.

b Percentage of EdU-labeled K562 cells in the presence or absence of α-amanitin. The gates indicate EdU-positive cells.

c Detection of γ-H2AX signals following α-amanitin treatment in early replicating K562 cells.

d Distribution pattern of early replication with α-amanitin treatment in K562 cells. The peaks of early replication are shown underneath (heavy grey). The empty triangles indicate early replication-induced pileups with 50 μg/mL α-amanitin treatment.

e Heatmaps of early replication signals (from EdU/HU) around non-TRs in mSBCs treated with the indicated concentrations of α-amanitin.

f Box plot showing the log2 ratio between the read number in non-TRs and that in TRs in the A compartments of mSBCs treated with the indicated concentrations of α-amanitin. The Wilcoxon Rank-sum test was applied for statistical analysis.

g Distribution pattern of early replication with DRB treatment in K562 cells. The empty triangles indicate early replication-induced pileups associated with the 30 μM DRB treatment.

h Schematic showing treatment with IAA to induce RNA polymerase II degradation in mESCs.

i Western blotting showing the amount of chromatin-bound MCM5 and H4 with or without RNA polymerase II degradation in mESCs in the G1 phase.

j Percentage of EdU-labeled mESCs with or without RNA polymerase II degradation. The red gates indicate EdU-positive cells.

k Heatmaps of early replication signals (from EdU/HU) around ERIZ-occupied non-TRs in wild-type mESCs treated with or without IAA.

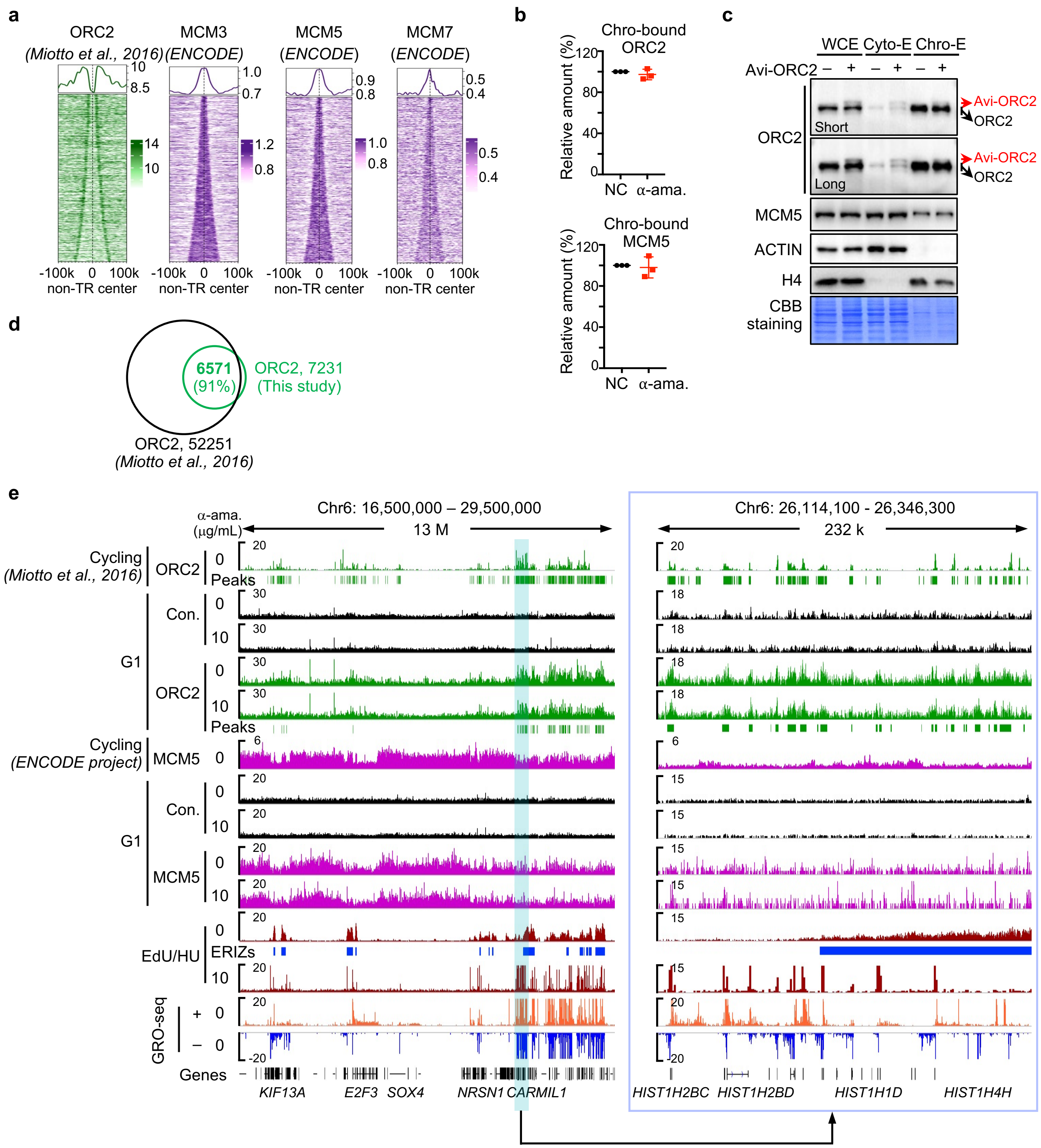

Fig. S5 Transcription inhibition does not lead to ORC2 relocation.

a The distribution patterns of ORC2, MCM3, MCM5, and MCM7 in cycling K562 cells. The ORC2 and MCM3/5/7 ChIP-seq data from the reference [31] and the ENCODE project, respectively, were re-analyzed. The non-transcribed regions (non-TRs) associated with ERIZs in K562 cells were used for analysis as described in Fig. 2**d**. Of note, ORC2 is shown with RPKM, while MCM is shown with fold-change. The data from ChIP-ed samples over the input samples are defined as fold-change data.

Fig. S5 Transcription inhibition does not lead to ORC2 relocation.

b The quantity of chromatin-bound ORC2 and MCM5. Each dot represents a biological replicate. The data are shown as the mean ± SD.

c Overexpression of Avi-ORC2 for ChIP-seq in G1-arrested K562 cells. K562 cells were transfected with plasmids expressing Avi-ORC2 and immediately incubated with CDK4/6 inhibitor palbociclib for 48 hours. The whole-cell extract (WCE), cytoplasmic extract (Cyto-E), and chromatin extract (Chro-E) were isolated for western blotting. The red arrow indicates Avi-ORC2, whereas the black arrow indicates endogenous ORC2. CBB, Coomassie brilliant blue.

d Venn diagram showing the overlapping ORC2 peak numbers between cycling and G1-arrested K562 cells.

e The distribution of ORC2, MCM5, and early replication initiation in the presence or absence of 10 μg/mL α-amanitin in G1-arrested K562 cells.

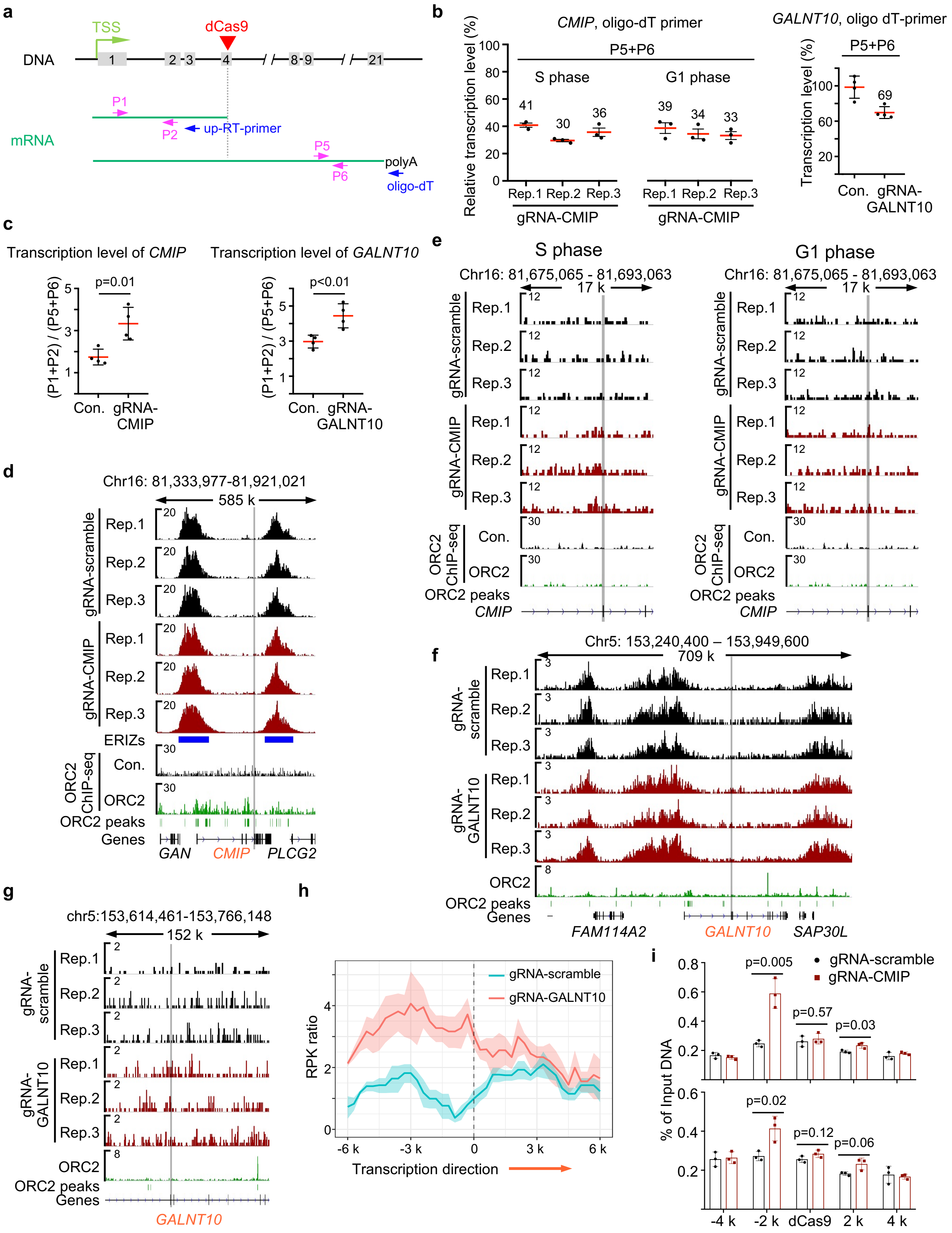

Fig. S6 dCas9-mediated transcription blockade induces MCM5 accumulation and replication initiation.

Fig. S6 dCas9-mediated transcription blockade induces MCM5 accumulation and replication initiation.

a Schematic showing the binding sites of primer sets applied to detect the transcription level of dCas9-targeted genes. The red triangle indicates the dCas9 binding sites. The blue arrows indicate the primers for reverse transcription, whereas the pink arrows indicate the primers used for qPCR detection.

b The transcription levels of *CMIP* and *GALNT10* in dCas9-treated K562 cells. Con., control; Rep., replicate. gRNA-CMIP or gRNA-GALNT10 represents samples treated with dCas9/gRNA targeting *CMIP* or *GALNT10*, respectively. The transcription level of *CMIP* is normalized to the level in the control sample, defined as the relative transcription level. Data are shown as the mean ± SD. The mean value is marked by the red line.

c The ratio of upstream transcription levels (P1+P2) over the downstream levels (P5+P6) in the dCas9-treated K562 cells. Con., control. gRNA-CMIP or gRNA-GALNT10 represents samples treated with dCas9/gRNA targeting *CMIP* or *GALNT10*, respectively. Data are shown as the mean (red line) ± SD.

d The patterns of ERIZs at the *CMIP* locus in dCas9-treated K562 cells with scrambled (black) or *CMIP* gRNA (red). The grey line indicates the binding sites of gRNA-CMIP. Biological replicates are indicated as Rep.1, Rep.2, or Rep.3.

e The distribution of early replication signals around dCas9-binding sites in early S phase (left) and G1-arrested (right) K562 cells. Legends are depicted as described in **d**.

f The patterns of early replication initiation at *GALNT10* in dCas9-treated K562 cells transfected with scrambled gRNAs (black) or gRNAs targeting *GALNT10* (red).

g Distribution of EdU/HU signals surrounding dCas9 binding sites in *GALNT10*. Legends are depicted as described in **f**.

h The normalized read density of EdU/HU signals within ±6 kb of the gRNA-GALNT10 binding sites in early S-phase K562 cells.

i The other two biological replicates of MCM5 ChIP-qPCR around the dCas9 binding sites on *CMIP*. Legends are depicted as described in Fig. 5**f**.

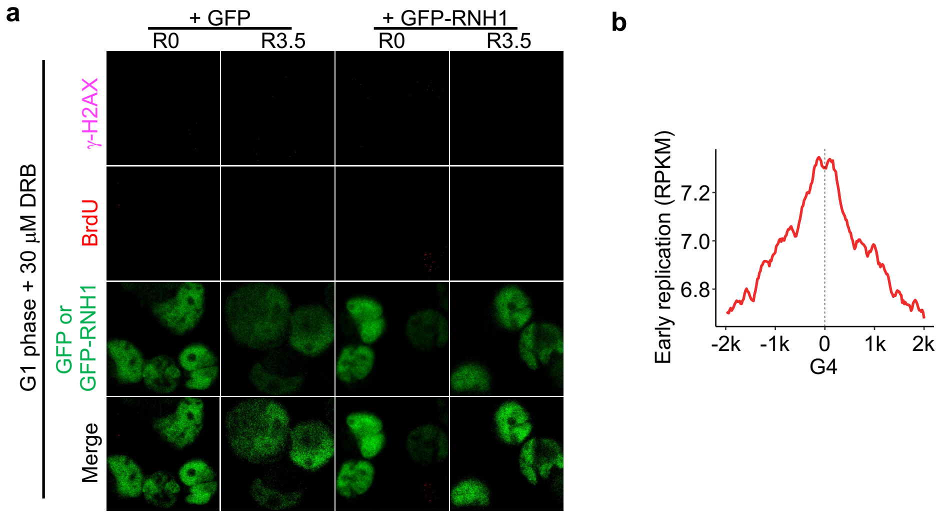

Fig. S7 Collisions of transcription and DNA replication initiation induces genome instability.

a Detection of γ-H2AX foci induced by DRB withdrawal with or without RNase H1 (GFP-RNH1) over-expression in G1 phase K562 cells. Legends are depicted as described in Fig. 6**d**.

b The distribution of early replication signals (from EdU/HU) at G-quadruplex (G4) sites in K562 cells. G4 ChIP-seq data are aligned at the center, indicated by a dashed line, and re-analyzed from [87].

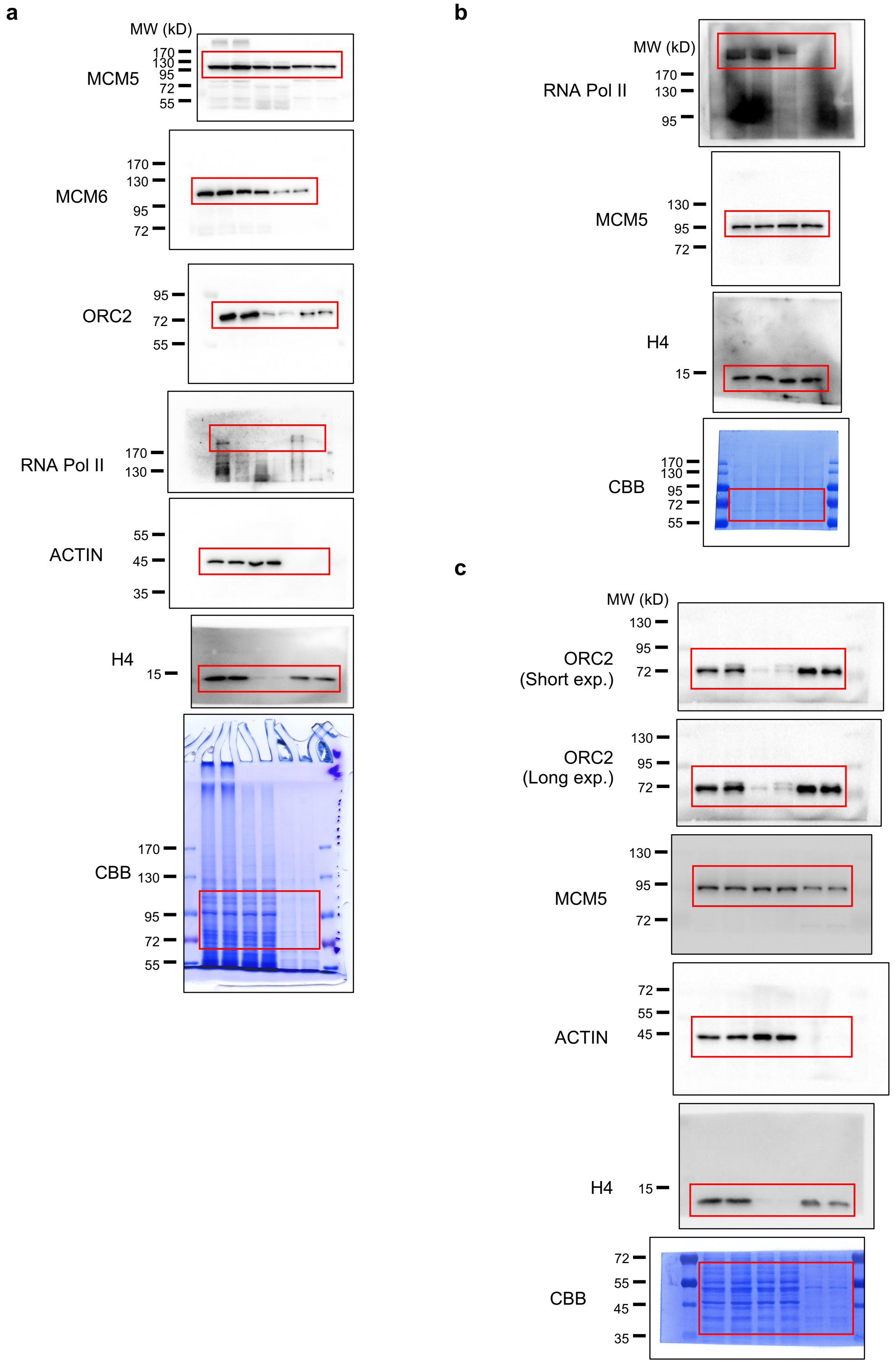

Fig. S8 The full uncropped pictures for Western blots.

a-c Uncropped Western blot data showed in Fig. 4**a** (**a**), Additional file 1: Fig. S4**i** (**b**), and Additional file 1: Fig. S5**b** (**c**). MW, molecular weight. The red boxes show regions presented in the assembled panels.
